# Development, Physicochemical Properties, and Antibacterial Activity of Propolis Microcapsules

**DOI:** 10.3390/foods12173191

**Published:** 2023-08-24

**Authors:** Qingya Zhang, Ao Yang, Weihua Tan, Wenchao Yang

**Affiliations:** 1 College of Animal Science (College of Bee Science), Fujian Agriculture and Forestry University, Fuzhou 350002, China; zhy1998122020@163.com (Q.Z.); ya104110@126.com (A.Y.); tanweihuayes@126.com (W.T.); 2 College of Food Science, Fujian Agriculture and Forestry University, Fuzhou 350002, China

**Keywords:** propolis microcapsule, spray-drying, micromorphology, antibacterial activity

## Abstract

Propolis is a well-known natural antibacterial substance with various biological activities, such as anti-inflammatory and antioxidant activity. However, applications of propolis are limited due to its low water solubility. In this study, propolis microcapsules were developed with a core material of ethanol extract of propolis and shell materials of gum arabic and β-cyclodextrin using a spray-drying technique. The optional processing formula, particle size distribution, morphology, dissolution property, and antibacterial activity of propolis microcapsules were determined. The results showed that the optional processing obtained an embedding rate of 90.99% propolis microcapsules with an average particle size of 445.66 ± 16.96 nm. The infrared spectrogram and thermogravimetric analyses showed that propolis was embedded in the shell materials. The propolis microcapsules were continuously released in water and fully released on the eighth day, and compared to propolis, the microcapsules exhibited weaker antibacterial activity. The minimum inhibitory concentrations (MICs) of propolis microcapsules against *Escherichia coli* and *Staphylococcus aureus* were 0.15 and 1.25 mg/mL, and their minimum bactericidal concentrations (MBCs) were 0.3 and 1.25 mg/mL, respectively. This water-soluble propolis microcapsule shows the potential for use as a sustained-release food additive, preservative, or drug.

## 1. Introduction

Propolis is processed by western worker bees after they collect resin from plant buds and plant secretions or stems, branches, and leaves of different plants and mix it with the secretions of their maxillary glands and wax glands. At present, there are more than 800 compounds in propolis, which represent compound classes of aliphatic acids and their esters, aromatic acids and their esters, flavonoids and other plant phenolics, terpenoids, carbohydrates, amino acids, vitamins (B_1_, B_2_, B_6_, C, and E), and minerals (aluminum, antimony, calcium, cesium, copper, iron, lanthanum, manganese, mercury, nickel, silver, vanadium, and zinc) [1]. The collection season, botanical source [2], age [3], solvent, and extraction process [4] influence the bioactive compounds of propolis. Propolis exhibits antibacterial, antiviral, and antioxidant activities as well as immune-stimulatory activities, which protect the honey bee colonies against microorganisms and parasites [5].

The antibacterial effect of propolis has been reported in many related studies by scholars. Propolis exhibits antibacterial activities against *Staphylococcus aureus*, *Escherichia coli* [6], *Streptococcus mutans*, *Streptococcus sobrinus*, *Actinomyces naeslundii* [2], *Pseudomonas aeruginosa* [7], *Bacillus subtilis*, *Listeria monocytogenes*, *Paenibacillus larvae* [8], *Micrococcus luteus* [9], and more than 600 bacterial strains [10]. Propolis also has antifungal activity against *Candida albicans*, *C. dubliniensis*, *C. glabrata*, *C. krusei*, *C. parapsisolis*, *C. tropicalis*, and *Saccharomyces cerevisiae* as well as against molds such as *Alternaria solani*, *Alternaria alternata*, *Aspergillus niger*, *Aspergillus ochraceus*, *Botrytis cinerea*, *Cladosporium* spp., *Fusarium solani*, *Fusarium oxysporum*, *Mucor mucedo*, *Penicillium digitatum*, *Penicillium expansum*, *Penicillium chrysogenum*, *Rhizopus stolonifera*, *Rhodotorula mucilaginosa*, and *Trichophyton* spp. [11,12]. The effective antibacterial properties of propolis lead to its widespread application, including applications in foods, functional foods, drugs, livestock, and cosmetics [13]. However, the low water solubility of propolis limits its application in many other areas.

Microcapsules, including nanocapsules, can modify the solubility of propolis. Microcapsules are an advanced processing technique whereby food components or functional constituents (core) are encapsulated inside a particular material (shell). Microcapsules perform several functions, including roles in miscibility, evaporation, reaction, stress, and controllable release [14]; as a result, diverse solutions are produced that can be applied to address challenges in the electronics, medicine, textile, cosmetic, chemical [9], and construction industries [15]. Various kinds of polymeric materials are employed as shell materials of microcapsules, such as polyurea formaldehyde, polyurethane, polymethyl methacrylate, melamine formaldehyde, polyaniline, and gelatin [16]. The technologies used to process microcapsules include spray-drying, freeze-drying, electrospinning/electrospraying, inclusion complexes, emulsification, liposomal systems, ionic gelation, and coacervation [17]. Spray-drying is widely applied to process microcapsules and exhibits several advantages, as it is rapid, versatile, cost-effective, and easy to scale up, and it exhibits high encapsulation efficiency, retention, and relatively good storage stability; however, it also involves nonuniform particles with a wide size distribution, active compound degradation, and loss of product [18].

The encapsulated flavonoids, lignans, terpenoids, essential oils, and other bioactive compounds obtained from herbs and spices have antibacterial effects. Essential oil microcapsules have an inhibition effect against *Penicillium digitatum*, *Colletotrichum gloesporioides*, *Colletotrichum brevisporum*, *Penicillium expansion*, *Botrytis cinereal*, and other pathogenic fungi, and have antibacterial activity against *Listeria monocytogenes*, *Salmonella typhimurium*, *S. aureus,* and *E. coli* pathogenic bacteria [19]. There are several reports about antibacterial, antioxidant, anti-inflammatory, and antimicrobial activities of salicylic acid microcapsules [20], *Elaeocarpus tectorius* (Lour.) Poir. leaf extract microcapsules [21], and other plant-derived extraction microcapsules [22]. They have been used for fruit and vegetable preservation [23].

In recent years, there have been some reports about the preparation of propolis microcapsule powder or propolis microparticle liquid. Propolis microcapsules/microparticles are developed using maltodextrin by spray-drying [24], isolated soy protein and pectin by freeze-drying [25], poly (ε-caprolactone) by emulsification–solvent evaporation techniques [26], and so on. These propolis microcapsules/microparticles were used as antimicrobial agents and anticancer material and in controlled-release drugs. In a few reports, propolis microcapsules were developed using gum arabic or/and β-cyclodextrin (β-CD), which exhibit chemical and physical stability, show the ability to form inclusion complexes with a wide variety of organic compounds, and increase the water solubility of various sparingly soluble compounds [17].

The main objective of this manuscript was, therefore, to develop an optional processing formula of propolis microcapsules using an orthogonal experiment based on the embedded rate of propolis by the spray-drying method. The particle size distribution, micromorphology, dissolution property, and antibacterial activity of propolis microcapsules were determined. The results can provide reference value for propolis microcapsules used as a sustained-release antibacterial agent, drug, or agent to preserve the freshness of foods.

## 2. Materials and Methods

### 2.1. Preparation of Propolis Extract

The extraction of raw poplar propolis, harvested in Qinglong Manchu Autonomous County, Qinhuangdao, Hebei, China, was performed as in our previous report [27] and modified. Briefly, raw propolis was extracted using ethanol (ratio (*w*/*v*) of propolis and 70% ethanol was 1:8). The solution was stirred for 2 h and treated with ultrasonic waves (40 kHz and 30 °C for 60 min). The mixture was filtered with filter paper (BS-TFP-110B, Labgic Technology Co., Ltd., Hefei, China). The supernatant was partially evaporated using a vacuum oven (DZF-6050, Shanghai Yiheng Scientific Instrument Co., Ltd., Shanghai, China) at 45 °C after centrifuging at 4000× *g* for 10 min (5810R, Eppendorf, Hamburg, Germany). Then, the extract was stored at 4 °C to solidify and remove beeswax on the surface. The extracted propolis was stored at −30 °C for further experimentation.

### 2.2. Optimal Formula for Propolis Microcapsule Powder Process

#### 2.2.1. Orthogonal Experiment

According to the reported method [28], β-CD (AR, Huaxing Biochemical Co., Ltd., Mengzhou, China) and gum arabic (AR, Shanghai Macklin Biochemical Technology Co., Ltd., Shanghai, China) were separately dissolved in 200 mL deionized water assisted by a mechanical mixer (RW 20 digital, IKA, Staufen, Germany). Then, they were mixed and homogenized for 30 min. After stewing for 20 min, 10% propolis ethanol (70%) solution, gum arabic, and β-CD solution were mixed and stirred for 1 h at 450 rpm and then ultrasonicated for 20 min. The stable propolis microcapsule emulsion for spray-drying was obtained after stewing for 12 h.

The propolis microparticle emulsion was dried by a small spray-dryer (JOYN-8000T, Shanghai Joyn Electronic Co., Ltd., Shanghai, China) under the following processing conditions: flow rate 10 mL/min, air pressure 3.2 kPa, nozzle diameter 1.5 mm, inlet temperature 135 °C, and outlet temperature 85 °C, according to the results of pre-experiments. Propolis microcapsule powder samples were obtained from collection bottles and stored in sealed bags at 4 °C for further analysis.

Based on the single-factor experimental results, 3-factor (ratio of shell materials, ratio of core to shell material, and homogenization rate) and 3-level orthogonal experiments were designed according to the indicator, which was the embedding rate of propolis in microcapsules, to optimize the process parameters (Table 1).

The optimal formula for processing the propolis microcapsule powder was verified with the measurement of the embedding rate in triplicate.

#### 2.2.2. Embedding Rate

The total flavonoid content (TFC) of propolis or propolis microcapsules was determined according to the report [29], using rutin as the standard substance and calculating the content of flavonoids according to the standard curve (y = 1.6112x + 0.0572, r^2^ = 0.9994).

The TFC in the propolis microcapsules was determined as follows. Propolis microcapsules 20 mg was added to 20 mL of 70% ethanol solution followed by ultrasonic treatment for 30 min. This solution was homogenized at 450 rpm for 10 min at 45 °C and diluted to 50 mL using ethanol solution. An amount of 1 mL prepared solution, 6 mL water, and 1 mL 5% sodium nitrite solution were uniformly mixed and stewed for 6 min. An amount of 1 mL of 10% aluminum nitrate solution was then uniformly mixed and stewed for 6 min. Then, 10 mL of 4.3% sodium hydroxide solution was added and diluted with water to 25 mL. The OD value (415 nm) was determined after uniform mixing and stewing for 15 min.

Determination of TFC on the surface of the propolis microcapsules was determined as follows. Propolis microcapsule powder 20 mg was added to 20 mL absolute ethanol. The solution was filtered using Grade 4 filter paper (Whatman, Hangzhou, China), and the filtrate was diluted to 50 mL. Then, 1 mL solution was treated in the same manner as the determination of total flavonoid content in propolis.

The embedding rate was calculated as Equation (1):

Embedding rate (%) = (TFC of propolis microcapsules − surface TFC of propolis microcapsules)/TFC of propolis microcapsules × 100(1)

### 2.3. Determinations of Characteristics of Propolis Microcapsules

#### 2.3.1. Micromorphology Determination of Propolis Microcapsules by Scanning Electron Microscopy (SEM)

The micromorphologies of propolis microcapsules and shell materials were observed by scanning electron microscopy (SEM, Zeiss Sigma 300, Aalen, Germany). Propolis microcapsule and shell materials were gold-plated in a vacuum environment for 90 s. Observation of acceleration voltage was from 10 kV to 15 kV.

#### 2.3.2. Determinations of Fourier Translation Infrared Spectroscopy (FT-IR) Spectrogram of Propolis, Shell Materials, and Propolis Microcapsules

Samples of propolis, shell materials, and propolis microcapsules were fully dried and mixed with potassium bromide (KBr) in a ratio of 1:60 (*w*:*w*), superlatively. The FT-IR was determined by Fourier infrared spectrometer (Thermo Scientific iN10, Waltham, MA, USA) at room temperature after samples were ground and tableted. The scanning range was 400–4000 cm^−1^ at a frequency of 2 cm^−1^. An average number of 40 scans were obtained from each sample.

#### 2.3.3. Determinations of Thermal Stability of Propolis, Shell Materials, and Propolis Microcapsules

The thermal stability of propolis, shell materials, and propolis microcapsules was determined using a thermogravimetric analyzer (Netzsch TG 209 F3 Tarsus, Selb, Germany). Approximately 3 mg of each sample was placed in an alumina crucible to start the program. The determination conditions were as follows: nitrogen flow rate 20 mL/min, temperature range 50–500 °C with increasing rate of 10 °C/min.

#### 2.3.4. Determinations of Agglomeration Degree, Wettability, Flowability, and Particle Size of Propolis Microcapsules

The agglomeration degree and flowability of the propolis microcapsules were determined according to the report [30].

The wettability of the propolis microcapsules was determined as follows. Accurately measured 0.1 g samples were scattered on the surface of 100 mL deionized water at room temperature (25 ± 1 °C) without stirring to be naturally wetted. The wetting time of the last particle was recorded as the wettability [31].

The particle sizes of the propolis microcapsules were determined using a nanoparticle size analyzer (Winner 803, Winner Particle Instrument Stock Co., Ltd., Jinan, China). The concentration of propolis microcapsules in water solution was 1 mg/mL. Then, 1 mL solution was diluted 100-fold with deionized water. The solution (2 mL) was employed for determination at 25 °C after balance for 60 s [32].

#### 2.3.5. Determinations of Sustained-Release Ability of Propolis Microcapsules

The sustained-release ability of the propolis microcapsules was determined [33]. The prepared propolis microcapsules (20 mg) were dissolved in 45 °C deionized water (30 mL), which was shaken 120 times, ultrasonicated for 30 min, and then diluted to 50 mL. The TFC of dilution was determined according to the earlier method. The released TFCs were measured on days 0, 2, 4, 6, and 8, respectively.

### 2.4. Determinations of Antibacterial Activity of Propolis Microcapsules

Bacterial strains of *S. aureus* (ATCC 6538, Gram-positive) and *E. coli* (ATCC 8739, Gram-negative) were purchased from the Guangdong Microbial Culture Collection Center, China. They were employed to determine the antimicrobial activity of the propolis microcapsules. The minimum inhibitory concentrations (MIC) and minimum bactericidal concentrations (MBC) of propolis microcapsules were determined according to the report [34]. Luria–Bertani (LB) broth medium (containing tryptone 10.0 g, yeast extract 5.0 g, and NaCl 10.0 g in 1 L water; FMB, Sangon Biotech, Shanghai, China) was prepared for further experiement. Propolis (0.100 g) was dissoved in 10 mL Ethylene glycol 400, and then 30 mL water and 60 mL sterilized LB broth medium were added and mixed evenly. Propolis microcapsules (0.2 g)was dissolved in 40 mL water, and then 60 mL sterilized LB broth medium was added and mixed evenly. And then they were gradient-diluted with sterilized LB broth medium. Different final concentrations of propolis microcapsules or propolis dilutions (0.05, 0.1, 0.15, 0.2, 0.25, and 0.3 mg/mL) and 1.5 × 10^8^ CFU *S. aureus or E. coli* were cultured at 37 °C for 24 h, and then OD600 was determined. MIC was calculated according to the OD values. Then, the cultured medium was uniformly coated on LB broth agar (containing tryptone 10.0 g, yeast extract 5.0 g, NaCl 10.0 g, and agar 15.0 g in 1 L; FMB, Sangon Biotech, Shanghai, China) to check the minimum concentration to kill 99.9% of the tested microorganisms (MBC). The higher concentrations of propolis microcapsules against *E. coli* were designed as 0.25, 0.5, 0.75, 1, 1.25, and 1.5 mg/mL because the MBC of the propolis microcapsules was higher than 0.3 mg/mL.

### 2.5. Data Analysis

All experiments were performed in triplicate. The experimental results were analyzed using GraphPad Prism 8.4.3 (GraphPad Software, Inc., San Diego, CA, USA) and Statistical Program for Social Sciences (SPSS 8.5, IBM, Armonk, NY, USA) for Windows and expressed as mean ± standard deviation. Differences among mean values of different groups were examined at the *p* < 0.05 significance level.

## 3. Results

### 3.1. Preparation of Propolis Microcapsules

The results of the 3-factor, 3-level orthogonal experiment are shown in Table 2.

The optimal formula A_2_B_3_C_1_ was the ratio of core to shell 1:2, homogenization rate 450 rpm, and the ratio of shell materials gum arabic to β-CD 2:1. The operating conditions of the small spray-dryer were as follows: flow rate 10 mL/min, air pressure 3.2 kPa, nozzle diameter 1.5 mm, and inlet and outlet temperatures 135 °C and 85 °C. The embedding rate reached 90.99%.

The optimal formula was verified through a validation experiment, in which the embedding rate was 91.86 ± 0.01%.

### 3.2. Characteristics of Propolis Microcapsules

#### 3.2.1. Scanning Electron Microscopy (SEM) of Propolis Microcapsules

**Figure 1:**
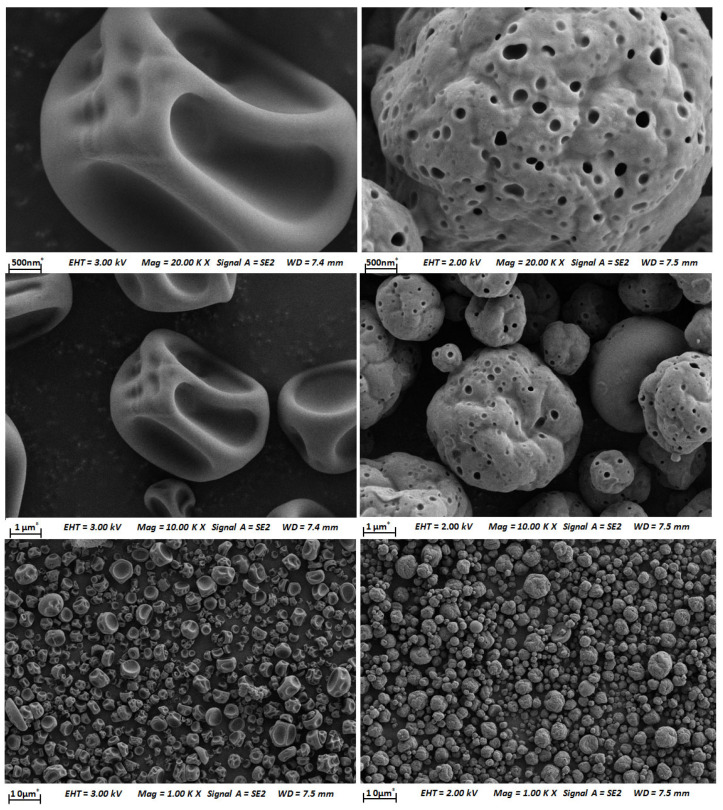
Micromorphology of shell materials (**left**) and propolis microcapsules (**right**).

The surface of the propolis microcapsule and the shell materials were scanned with a high-energy beam of electrons in a raster scan pattern. The micromorphology of the samples is shown in Figure 1.

The propolis microcapsule is a light-yellow powder without an obvious propolis smell. The micromorphology of propolis microcapsules showed that propolis is filled in the cavity of shell materials. And the propolis microcapsules have a spherical shape with a smooth surface (Figure 1).

#### 3.2.2. The Analysis of Fourier Translation Infrared Spectroscopy (FT-IR) of Propolis Microcapsules

**Figure 2:**
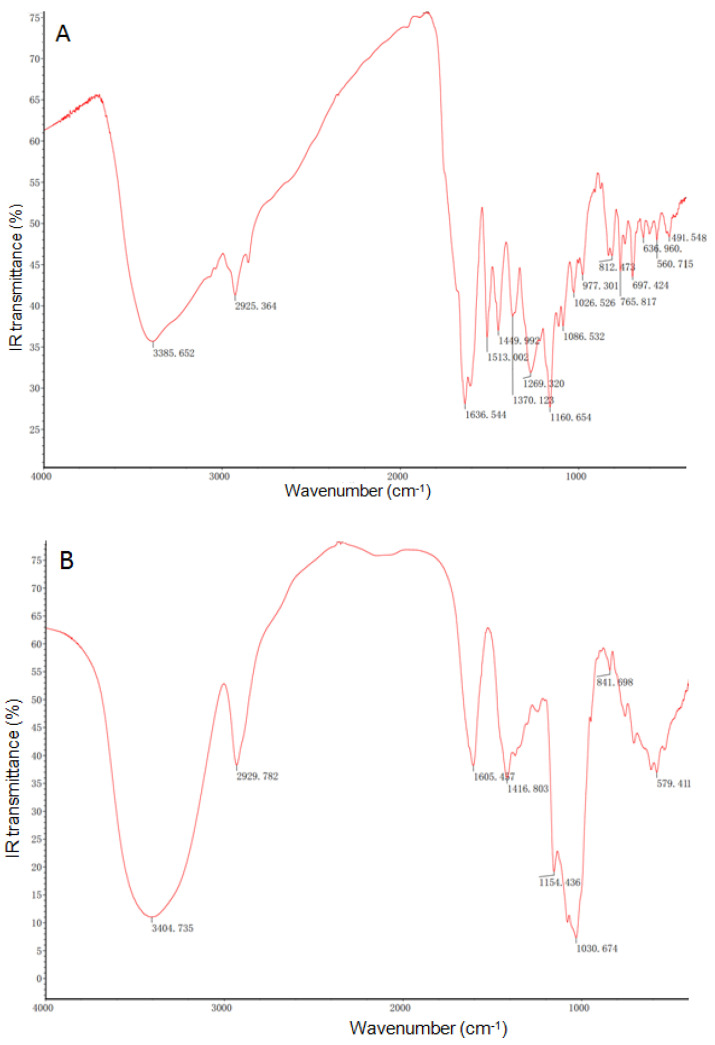

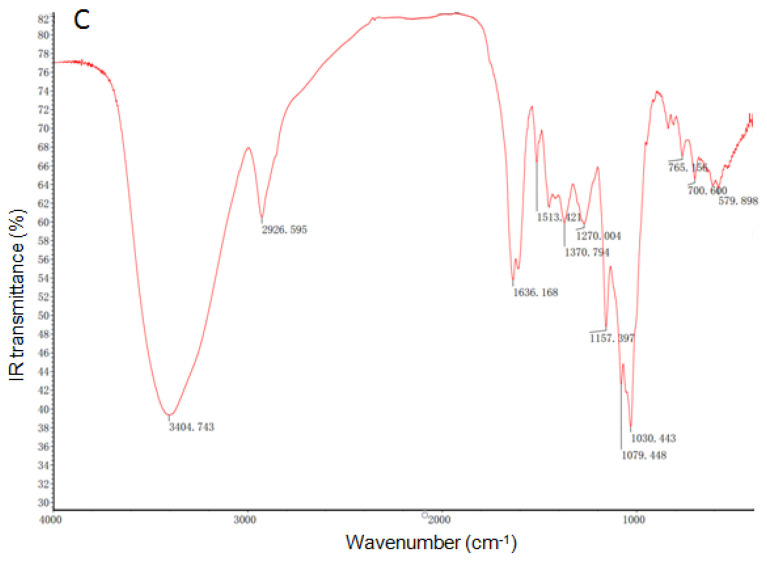
The FT-IR spectrogram of propolis (**A**), shell material (**B**), and propolis microcapsules (**C**).

The FT-IR analysis showed that there were the existence of or changes in functional groups and chemical bonds among propolis, shell material, and propolis microcapsules (Figure 2). The FT-IR spectrogram showed that peaks at 1636, 1513, 1370, 1270, and 765 cm^−1^ were recorded in both propolis and propolis microcapsules but not in shell materials, which means that components containing alkenyl C=C stretch olefinic (alkene), aromatic nitro compounds, nitrate ion/aliphatic nitro compounds, organic phosphates (P=O stretch), and aliphatic chloro compounds (C-Cl stretch) in propolis are embedded. Some peaks at 3385, 2925, 1160, 1026, and 697 cm^−1^ in the FT-IR spectra of propolis shifted compared with the microcapsules, which indicated that particular polyphenols or flavonoids, such as alcohol and hydroxy compounds, and functional bound compounds containing methylene, C=C-C aromatic ring stretch, aromatic C-H in-plane bend, and C-H monosubstitution (phenyl) were involved in the formation of microcapsules [35].

#### 3.2.3. Thermal Stability of Propolis Microcapsules

**Figure 3:**
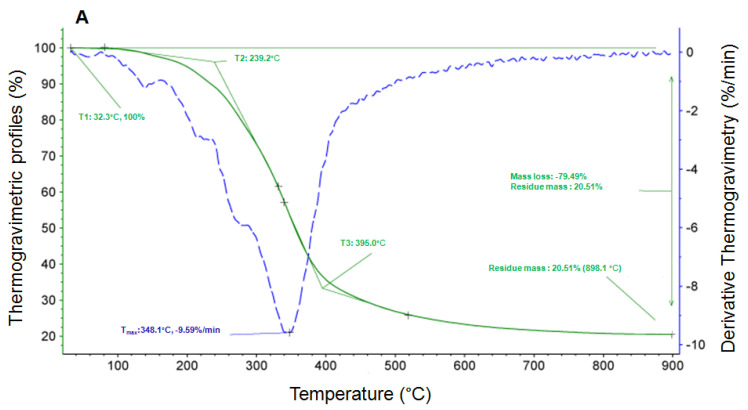

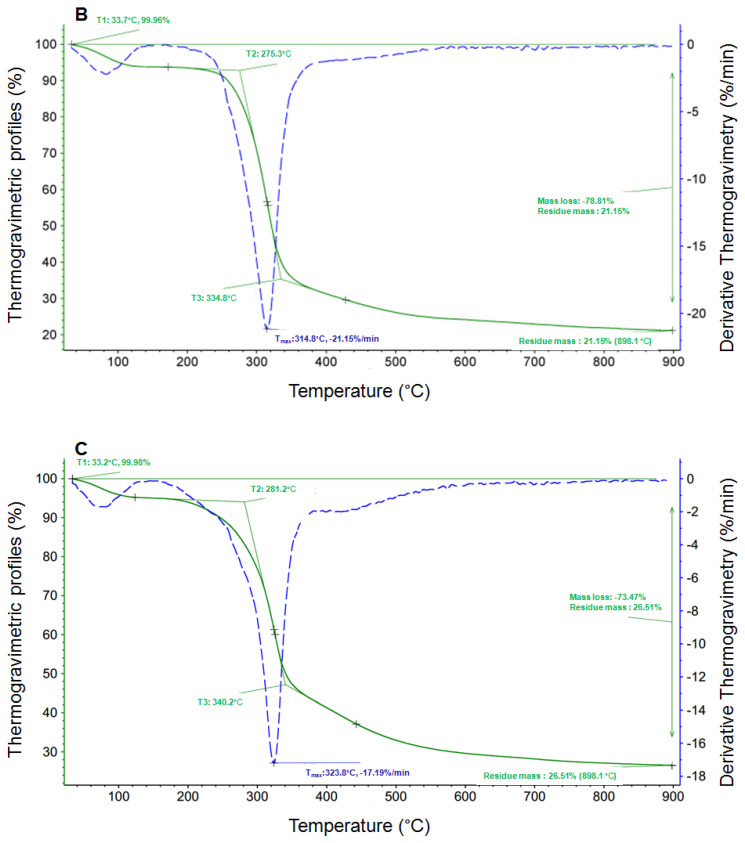
The thermogravimetric spectrogram of propolis (**A**), shell material (**B**), and propolis microcapsules (**C**). T1 means the onset temperature of the 1st phase; T2 means the end of 1st phase and the onset temperature of the 2nd phase; and T3 means the end of the 2nd phase and the onset temperature of the 3rd phase. T_max_ means the temperature at the highest derivative thermogravimetry.

There were three phases in the quality loss process (Figure 3). The onset temperature of the first phase; the end of the first phase and the onset temperature of the second phase; and the end of the second phase and the onset temperature of the third are listed in Table 3. The data showed that the propolis microcapsules are more stable at different temperatures than propolis.

#### 3.2.4. The Agglomeration Degree, Wettability, Flowability, and Particle Size of Propolis Microcapsules

The characteristics of propolis microcapsule powder are shown in Table 4.

**Figure 4:**
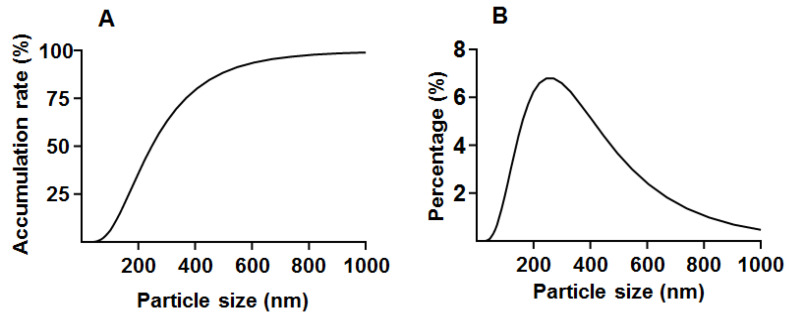
The particle sizes of propolis microcapsules ((**A**): accumulation rate; (**B**): percentage).

The average particle size of propolis microcapsules was 445.66 ± 16.96 nm with D_50_ 246.13 and D_90_ 523.66 nm. The accumulation rate and percentage of propolis microcapsules under different particle sizes are shown in Figure 4.

### 3.3. The Sustained-Release Ability of Propolis Microcapsules

**Figure 5:**
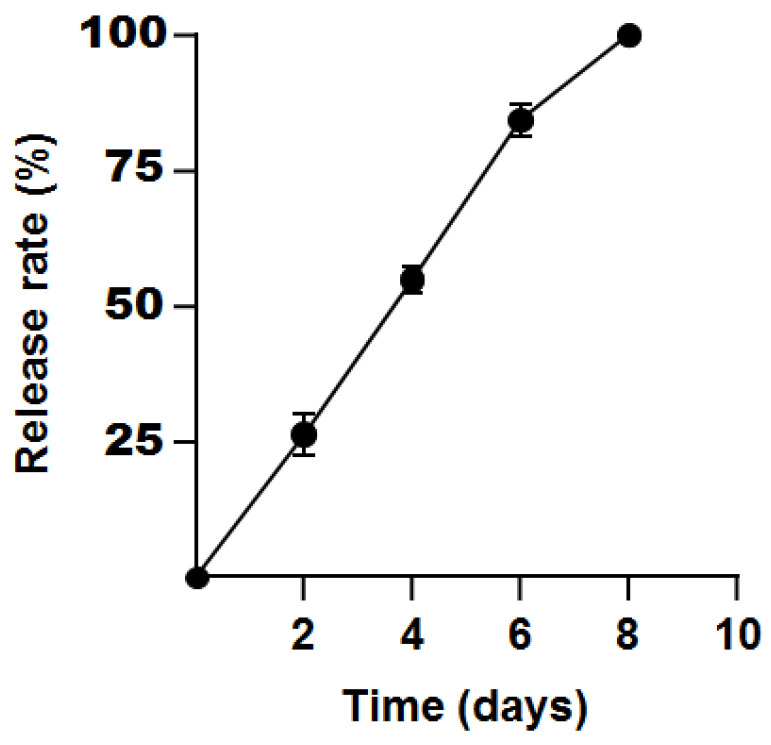
The sustained release of propolis microcapsules.

The sustained-release ability of propolis microcapsules is shown in Figure 5. The release amount of propolis was almost linear with time and was totally released after 8 days.

### 3.4. Antibacterial Activity of Propolis Microcapsules

The antibacterial activity (minimum inhibitory concentration, MIC; minimum bactericidal concentration, MBC) of propolis microcapsules was weaker than that of propolis (Table 5).

## 4. Discussion

Propolis microcapsules were developed using gum arabic and β-CD as shell materials in the spray-drying technique. Propolis was well embedded, with a diameter of propolis microcapsule less than 1000 nm with an average of 445.66 ± 16.96 nm. Propolis microcapsules showed sustained-release properties and weaker antibacterial activity than propolis.

To process the propolis microcapsules, gum arabic and β-CD were designed as shell materials due to their suitable cavity size and low cost. Gum arabic has been widely used as a shell material due to its high encapsulation efficiency, water solubility, and stability against pH (2–8) and temperature (30–90 °C) [36]. Citrus flavonoid microcapsules [37], moxa oil microcapsules [38], propolis microcapsules [39], thyme essential oil microcapsules, and other microcapsules are prepared with gum arabic and/or other shell materials. β-CD is the most common natural cyclodextrin for the preparation of microcapsules [40,41] due to the high stability of many volatile aroma compounds bound to β-CD [42]. Essential oils of resveratrol, cinnamaldehyde, zanthoxylum bungeanum, eugenol, thymol, catnip, peppermint, oregano, zanthoxylum bungeanum resin, lavender, apple fragrance, neroline, jeringau rhizome, and rosemary nano/microcapsules are prepared with β-CD [43].

The ratio of propolis to shell materials was the most important factor for the embedding rate of propolis, and the optional ratio of propolis and shell materials was 1:2. In general, β-CD and core ratios of 1:1, 2:1, 1:2, and 2:2 can be developed depending on the size of the core and experimental conditions [44]. In the present experiment, the ratio of β-CD to propolis (core) was 2:3, which is reasonable because gum arabic was also added as a shell material. The presence of methyl groups [42,45] and the hydrophobic properties of the core or host can affect the cavity size and binding ability of β-CD [46], but the host–guest complexation mechanism has not been sufficiently elucidated [43].

Currently, β-CD microcapsules can be prepared by stirring, kneading, recrystallization, ultrasonication, and sol-gel methods, from which powder can be obtained by freeze-drying, spray-drying, and freeze-spray-drying techniques [43]. In this experiment, propolis microcapsules were processed by the spray-drying technique, which is a widely applied method to process nano/microcapsules [18]. This encapsulation method involves the preparation of a stable emulsion, homogenization of the dispersion, atomization of the emulsion, and dehydration of the atomized particles. Therefore, several parameters of the encapsulation process, including inlet and outlet temperatures, total solids, humidity, and the type of shell materials, significantly affect the quality of the final nanocapsule powder [47], and optimal parameters vary for different spray-drying machines.

The particle size is an important quality of the nanocapsule powder. The particle size of propolis microcapsules varies depending on the shell material, processing technique, and propolis type. The particle size of micro- and nano-Indonesian propolis was 3 µm to 300 nm, which was encapsulated by casein micelles with a homogenizer followed by sonication and a micro- and ultrafiltration system [48]. The particles of nanopropolises (Ag and Se) prepared by the green synthesis method were between 252 and 530 nm in size [49]. The minimum particle sizes of nanoparticles prepared using ultrasonication, self-assembly methods, hydrothermal methods, UV irradiation, and microwave irradiation were 94.89, 98.68 nm, 110 nm, 132.9 nm, and 158.6 nm, respectively. This indicates that high-temperature processing will increase the particle size [50]. In the present research, the average particle size of propolis microcapsules was 445.66 ± 16.96 nm, and 50% of the particles were under 200 nm. The larger particle size may result from the thermal treatment of spray-drying, liquid droplet size, and shell material concentration [51,52].

The characteristics of micro/nanocapsule powder were also determined by SEM, FT-IR, and thermogravimetric techniques. The present propolis microcapsules exhibited a spherical shape with a smooth surface, as shown in SEM images (Figure 1), which corresponds with selenium nanopropolis [53], silver nanopropolis [54], and nanopropolis encapsulated in gum arabic and maltodextrin [55]. The FT-IR spectrogram showed that peaks at 1636, 1513, 1370, 1270, and 765 cm^−1^ were recorded in propolis and propolis microcapsules but not in shell materials (Figure 2). According to the reference, the peak at 1636 cm^−1^ is attributed to flavonoids and/or amino acids with ν (C=O), ν (C=C), and δ_as_ (N-H) groups and vibration modes, and the peak at 1513 cm^−1^ is attributed to flavonoids and/or an aromatic ring with ν (aromatic) group and vibration mode. The peak at 1369 cm^−1^ is CH3 and/or flavonoids, which contain a δ_a_ (C-H) group and vibration mode. The peak at 1270 cm^−1^ contains ν (O-H) and/or δ_as_ (C-CO) groups and vibration modes. The poplar propolis also has peaks at 765 and 700 cm^−1^, which are alcohols and phenols with γ (O-H) groups and vibration modes [56]. The shifted peaks indicate that particular polyphenols or flavonoids are involved in the formation of nanoparticles, which was also observed with other kinds of nanopropolis [57]. The thermal stability results showed a higher end point of the second phase of the propolis microcapsule than that of the shell materials (Figure 3). This was also found in the thermogravimetric analysis of sodium alginate–green propolis extract–SiO_2_ film [58], nanopropolis in polymeric matrices [55], and membranes impregnated with nanoparticles loaded with propolis [59].

The agglomeration degree, wettability, and flowability are also important indices for the quality of the nanocapsule powder. The degree of nanoparticle agglomeration can reveal the nanoparticles’ useful properties. This characteristic can be influenced by drying methods, -OH groups present on the surface of nanoparticles, surface roughness, and storage [60,61,62,63]. The prepared propolis microcapsules exhibited good wettability because the wetting times needed were less than 60 s. Small particle size would increase the wettability, which might result from the increasing surface area of microcapsules that can interact with water [64,65]. Microcapsules prepared from acacia gum and β-CD containing a large number of hydrophilic groups will exhibit better wettability, which may contribute to their solubility [66]. Flowability is a distinguishing trait but not an inherent and comprehensive property of powder [67], and flowability was related to the equipment and the testing method used to describe the complex behavior of powder when it was mobilized or subjected to stress [68,69]. Flowability also has a major influence on the processability of powder [69]. The HR of the propolis microcapsule was 1.23, indicating that the flowability was fair [70]. This propolis microcapsule can be further processed into tablets or granules.

All the characteristics of propolis microcapsules mentioned above influence its activities, including the antibacterial activity. Compared to propolis, propolis microcapsules exhibited weaker antibacterial activity against *E*. *coli* and *S*. *aureus*. This was also found in gold nanoparticles of Brazilian red propolis (BRP) extract, in which MIC or MBC against *S. aureus*, *E. coli*, *S. mutans*, and *Candida albicans* were higher than those of BRP extract [71]. Poly (lactic-co-glycolic acid) nanoparticles containing red propolis hydroethanolic extract showed weaker antibacterial activity against *S. aureus* methicillin-resistant or methicillin-sensitive strains and *P. aeruginosa* than did propolis [72]. The propolis microcapsules presented inhibitory activity for *S. aureus* around 200–400 μg/mL and that of free propolis around 50–100 μg/mL [25]. Red propolis microcapsules presented an MIC of 135.87–271.74 μg/mL for *S. aureus* and an MIC of 271.74–543.48 μg/mL for *P. aeruginosa*, while free propolis displayed activity against them in all the concentrations studied (100 and 2000 μg/mL) [73]. It was also reported that the red propolis extract showed inhibition halos greater than those of chitosanate loaded with propolis [74]. This resulted from the lower propolis quantity and gradual release of propolis microcapsules (Figure 5). But a different result was reported when the inhibitory zone of propolis microcapsules, prepared with almond gum (AG) and sodium caseinate, increased from 18.22 to 23.60 mm for *S. aureus* and from 8.55 to 15.14 mm for *E. coli* [75]. The total sustained release of this propolis microcapsules was 8 days, which is shorter than that of propolis-embedded zeolite nanocomposites and propolis-based chitosan varnishes [76,77]. The release time of propolis microcapsules was different due to the different shell materials, solutions types and pH values, particle sizes, process techniques, and temperatures [55,76,77,78,79].

## 5. Conclusions

Propolis microcapsules were developed using gum arabic and β-CD as shell materials, with a diameter of less than 1000 nm and an average of 445.66 ± 16.96 nm. These water-soluble propolis microcapsules exhibit sustained-release properties and antibacterial activity, and can be developed as a sustained-release food additive, preservative, or drug.

## Figures and Tables

**Table 1:** The orthogonal experiment of propolis microcapsule process.

Level	Factors		
	Ratio of Gum Arabic to β-CD	Ratio of Core to Shell Material	Homogenization Rate (rpm)
1	1:1	1:1	450
2	2:1	1:1.5	550
3	1:2	1:2	650

**Table 2:** The results of the orthogonal experiment.

#	Factors			Indicator Embedding Rate (%)
	A (Ratio of Shell Materials)	B (Core:Shell)	C (Homogenization Rate)	
1	1	1	1	14.12
2	1	2	2	44.83
3	1	3	3	30.22
4	2	1	2	16.67
5	2	2	3	46.11
6	2	3	1	90.99
7	3	1	3	3.73
8	3	2	1	49.50
9	3	3	2	54.22
K_1j_	89.17	34.52	154.61	
K_2j_	153.77	140.44	115.72	
K_3j_	107.45	175.43	80.06	
$\bar{K_{1j}}$	29.72	11.51	51.54	
$\bar{K_{2j}}$	51.26	46.81	38.57	
$\bar{K_{3j}}$	35.82	58.48	26.69	
Rj	64.60	140.91	74.55	
Factor priority	B > C > A			
Optimal formula	A_2_B_3_C_1_			

**Table 3:** The different phases of propolis, shell material, and propolis microcapsules.

	The Onset of the 1st Phase	The End of the 1st Phase and the Onset of the 2nd Phase	The End of the 2nd Phase and the Onset of the 3rd Phase	Final Residue Mass (%)
Propolis	32.3	239.2	395	20.51
Shell material	33.7	275.3	334.8	21.15
Microcapsules	33.2	281.2	340.2	26.51

**Table 4:** The characteristics of propolis microcapsule powder.

Agglomeration Degree	Wettability	Bulk Density	Tapped Density	Hausner Ratio	Carr Index
3.62 ± 0.004%	55 ± 2 s	0.21 ± 0.01 g/cm^3^	0.26 ± 0.02 g/cm^3^	1.23 ± 0.02	18.45 ± 1.13%

**Table 5:** Antibacterial activity of propolis and propolis microcapsules.

	*Escherichia coli*		*Staphylococcus aureus*	
	Propolis	Propolis Microcapsules	Propolis	Propolis Microcapsules
MIC (mg/mL)	0.25	1.25	0.10	0.15
MBC (mg/mL)	0.25	1.25	0.25	0.30

## Data Availability

Data are contained within this article.
